# UAV RGB, thermal infrared and multispectral imagery used to investigate the control of terrain on the spatial distribution of dryland biocrust

**DOI:** 10.1002/esp.5189

**Published:** 2021-08-10

**Authors:** Javier Blanco‐Sacristán, Cinzia Panigada, Rodolfo Gentili, Giulia Tagliabue, Roberto Garzonio, M. Pilar Martín, Mónica Ladrón de Guevara, Roberto Colombo, Thomas P. F. Dowling, Micol Rossini

**Affiliations:** ^1^ Remote Sensing of Environmental Dynamics Lab University of Milano‐Bicocca Milan Italy; ^2^ Environmental remote sensing and spectroscopy laboratory (SpecLab) Spanish National Research Council (CSIC) Madrid Spain; ^3^ Universidad Rey Juan Carlos Móstoles Spain; ^4^ Centre for Ecological Research and Forestry Applications, CREAF‐CSIC‐UAB Barcelona Spain; ^5^ United Nations Environment Programme World Conservation Monitoring Centre Cambridge UK

**Keywords:** apparent inertia, thermal, biocrusts, biological soil crusts, drylands, lichen, moss, multispectral, thermal, UAV

## Abstract

Biocrusts (topsoil communities formed by mosses, lichens, bacteria, fungi, algae, and cyanobacteria) are a key biotic component of dryland ecosystems. Whilst climate patterns control the distribution of biocrusts in drylands worldwide, terrain and soil attributes can influence biocrust distribution at landscape scale. Multi‐source unmanned aerial vehicle (UAV) imagery was used to map and study biocrust ecology in a typical dryland ecosystem in central Spain. Red, green and blue (RGB) imagery was processed using structure‐from‐motion techniques to map terrain attributes related to microclimate and terrain stability. Multispectral imagery was used to produce accurate maps (accuracy > 80%) of dryland ecosystem components (vegetation, bare soil and biocrust composition). Finally, thermal infrared (TIR) and multispectral imagery was used to calculate the apparent thermal inertia (ATI) of soil and to evaluate how ATI was related to soil moisture (*r* ^2^ = 0.83). The relationship between soil properties and UAV‐derived variables was first evaluated at the field plot level. Then, the maps obtained were used to explore the relationship between biocrusts and terrain attributes at ecosystem level through a redundancy analysis. The most significant variables that explain biocrust distribution are: ATI (34.4% of variance, *F* = 130.75; *p* < 0.001), Elevation (25.8%, *F* = 97.6; *p* < 0.001), and potential solar incoming radiation (PSIR) (52.9%, *F* = 200.1; *p* < 0.001). Differences were found between areas dominated by lichens and mosses. Lichen‐dominated biocrusts were associated with areas with high slopes and low values of ATI, with soil characterized by a higher amount of soluble salts, and lower amount of organic carbon, total phosphorus (P_tot_) and total nitrogen (N_tot_). Biocrust‐forming mosses dominated lower and moister areas, characterized by gentler slopes and higher values of ATI with soils with higher contents of organic carbon, P_tot_ and N_tot_. This study shows the potential to use UAVs to improve our understanding of drylands and to evaluate the control that the terrain has on biocrust distribution.

## Graphical abstract

We used in synergy multi‐source UAV‐based imagery to map and study biocrusts’ ecology in a typical dryland ecosystem in central Spain. We processed RGB imagery by means of Structure from Motion techniques to map terrain attributes related to microclimate and terrain stability. We used thermal infrared imagery to calculate the apparent thermal inertia (ATI) of soils and to estimate how ATI was related to soil moisture. Finally, we used multispectral imagery to produce accurate maps of dryland ecosystem components (i.e., vegetation, bare soil and biocrust composition). We integrated traditional field approaches with new methodologies to explore the relationship between biocrusts and terrain attributes at landscape level. The very fine spatial resolution maps provided by UAVs were essential for a detailed description and monitoring of these complex and threatened ecosystems.

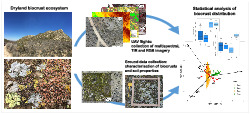

## 1 INTRODUCTION

Drylands constitute one of the largest biomes on Earth, covering ~47% of the terrestrial surface (Koutroulis, 2019). In these environments, topography determines the redistribution of scarce precipitation, controls water content and the availability of soil nutrients and organic matter affected by runoff (e.g., Aguiar & Sala, 1999; Manzoni et al., 2006; Puigdefábregas & Sánchez, 1996; Puigdefábregas et al., 1999; Puigdefábregas, 2005). This modifies not only vegetation distribution, but also the allocation of components that appear in plant interspaces, such as biocrust communities (Rodríguez‐Caballero et al., 2019). Biocrusts are a combination of topsoil communities including mosses, lichens, liverworts, bacteria, fungi, algae and cyanobacteria. They play an important role in nutrient cycling (Elbert et al., 2012; Weber et al., 2015), soil carbon (C) fluxes (Castillo‐Monroy et al., 2011; Tucker et al., 2019) and runoff/runon dynamics (Chamizo et al., 2012a; Faist et al., 2017; Rodríguez‐Caballero et al., 2015), playing a key role in dryland ecosystems' service maintenance (Rodríguez‐Caballero, Escribano, et al., 2017a; Rodríguez‐Caballero, Paul, et al., 2017b). The effect that these communities have on dryland surfaces depends on the dominant and developmental stage of the crust (Belnap & Lange, 2013; Concostrina‐Zubiri et al., 2013; Faist et al., 2017; Tucker et al., 2019). For example, biocrusts can increase water availability for plants by augmenting water retention in topsoil (Eldridge et al., 2020) and reducing soil evaporation (e.g., Adessi et al., 2018; Chamizo et al., 2016). They can also modify the erosion rate (Gao et al., 2020), which affects sediment accumulation, C and nutrient content in soils (e.g., Chamizo et al., 2017) and surface roughness (e.g., Rodríguez‐Caballero et al., 2015; Wang et al., 2017). For this reason, mapping the spatial patterns of different biocrust‐dominated surfaces and their extent is important to understand their role in the ecosystem.

The heterogeneous, mixed structure of dryland ecosystems is a challenge for biocrust mapping using remotely‐sensed imagery (Smith et al., 2019). Previous studies have used unmanned aerial vehicles (UAVs) to investigate dryland vegetation (e.g., Cunliffe et al., 2016; Sankey et al., 2018; Milling et al., 2018), proving that this is an achievable task. However, remote sensing of biocrusts has traditionally been relegated to other platforms, such as airborne (e.g., Weber et al., 2008; Rodríguez‐Caballero et al., 2014) and satellite sensors (e.g., Panigada et al., 2019). Only recently the potential of red, green and blue (RGB) imagery acquired from UAVs to identify dryland biocrusts has been explored (Havrilla et al., 2020; Jung et al., 2020; Sevgi et al., 2020). As biocrusts are difficult to distinguish from the background due to their small size and similar optical properties to vegetation and soil (Weber & Hill, 2016; Smith et al., 2019), UAVs integrated with multispectral cameras could greatly improve their identification. Multispectral sensors, in fact, usually have at least one spectral band in the red region at ~660–680 nm, that corresponds to the chlorophyll‐*a* absorption feature which is present in all chlorophytic biocrusts (Weber & Hill, 2016). Several authors (e.g., Blanco‐Sacristán et al., 2019; Panigada et al., 2019; Rodríguez‐Caballero, Escribano, et al., 2017a; Rodríguez‐Caballero, Paul, et al., 2017b; Román et al., 2019) have exploited the subtle differences in this absorption features for biocrust identification using the continuum removal (CR) algorithm (Clark & Roush, 1984). This allows for the normalization of reflectance to a common baseline, which enables the analysis of individual absorption features.

UAVs have been commonly used to obtain digital surface models (DSMs) from RGB imagery using digital photogrammetry, from which digital terrain models (DTMs) of a very fine spatial resolution can be derived. This information can be used to calculate topography‐related variables (Westoby et al., 2012). Detailed DTMs allow reseachers to apply hydrological models and therefore to study the impact of changing terrain properties on a landscape's hydrology (e.g., Lucieer et al., 2014). Thus, using these models it is possible to study the components of hydrological systems, such as surface and subsurface flows, which are key to understanding nutrient and sediment transport in the landscape (Stieglitz et al., 2003). However, site‐specific relationships between soil physical properties and topography generate deviations from larger‐scale climate patterns, which favours the formation of a heterogeneous community assembly (Rossi et al., 2014). In this context, as soil physical properties are a significant control on biocrust distribution in drylands (Bowker et al., 2016; Rodríguez‐Caballero et al., 2019), it is important to explore the link between DTM‐derived variables and soil properties. However, not all physical soil properties can be evaluated through DTMs. Key variables for biocrust development, such as soil moisture, need additional data sources in order to be estimated.

The knowledge of soil moisture distribution at a high spatio‐temporal resolution plays an important role in the understanding of ecological and hydrological processes at the basin scale. Soil moisture plays a key role in surface water flow by controlling transport processes in the soil–plant–atmosphere system (Campbell & Norman, 1988). However, estimation of the soil moisture involves in situ measurements by standard point‐based techniques (e.g., thermogravimetric method or time domain reflectometry) and requires interpolation techniques to obtain spatially explicit information. Different methods have been applied to analyse the spatial distribution of soil moisture based on thermal inertia, a physical property of the surfaces, which determines resistance to temperature change under seasonal and diurnal heating (Price, 1985; Short & Stuart, 1982). The dynamics of soil thermal properties and water content have also been analysed using proximal sensing (Krzeminska et al., 2012; Nearing et al., 2012; Negm et al., 2017). In arid and semi‐arid environments, the spatial distribution of soil surface water content in bare soils has been evaluated from high resolution visible/near‐infrared (VIS/NIR) and thermal infrared (TIR) airborne and satellite data (Van Doninck et al., 2011) through the computation of the apparent thermal inertia (ATI). However, the coupling between ATI and soil moisture is not straightforward in heterogeneous surfaces since ATI might be directly related to soil moisture only in homogeneous areas with a single land cover type, with limitations observed for vegetated surfaces (Maltese et al., 2013; Van Doninck et al., 2011). For these reasons, the synergistic use of TIR and multispectral cameras on board UAVs allows the derivation of high spatial resolution maps of ATI, with the possibility to mask the vegetation component, helping to decipher the problem of mixed surfaces.

The objective of this study was to evaluate the effect of terrain attributes in the distribution of dryland ecosystem components' using multisensor UAV‐based imagery by( i) mapping dryland constituents (i.e., vegetation, biocrusts, and bare soil) through multispectral imagery; (ii) estimating terrain attributes from structure‐from‐motion (SfM) techniques applied to RGB imagery and evaluating their relation with soil properties; (iii) estimating soil moisture using maps of ATI derived from TIR imagery; (iv) evaluating through a multivariate statistical approach how terrain attributes and soil moisture affect biocrust distribution in the study area.

## 2 MATERIALS AND METHODS

### 2.1 Study area

**FIGURE 1:**
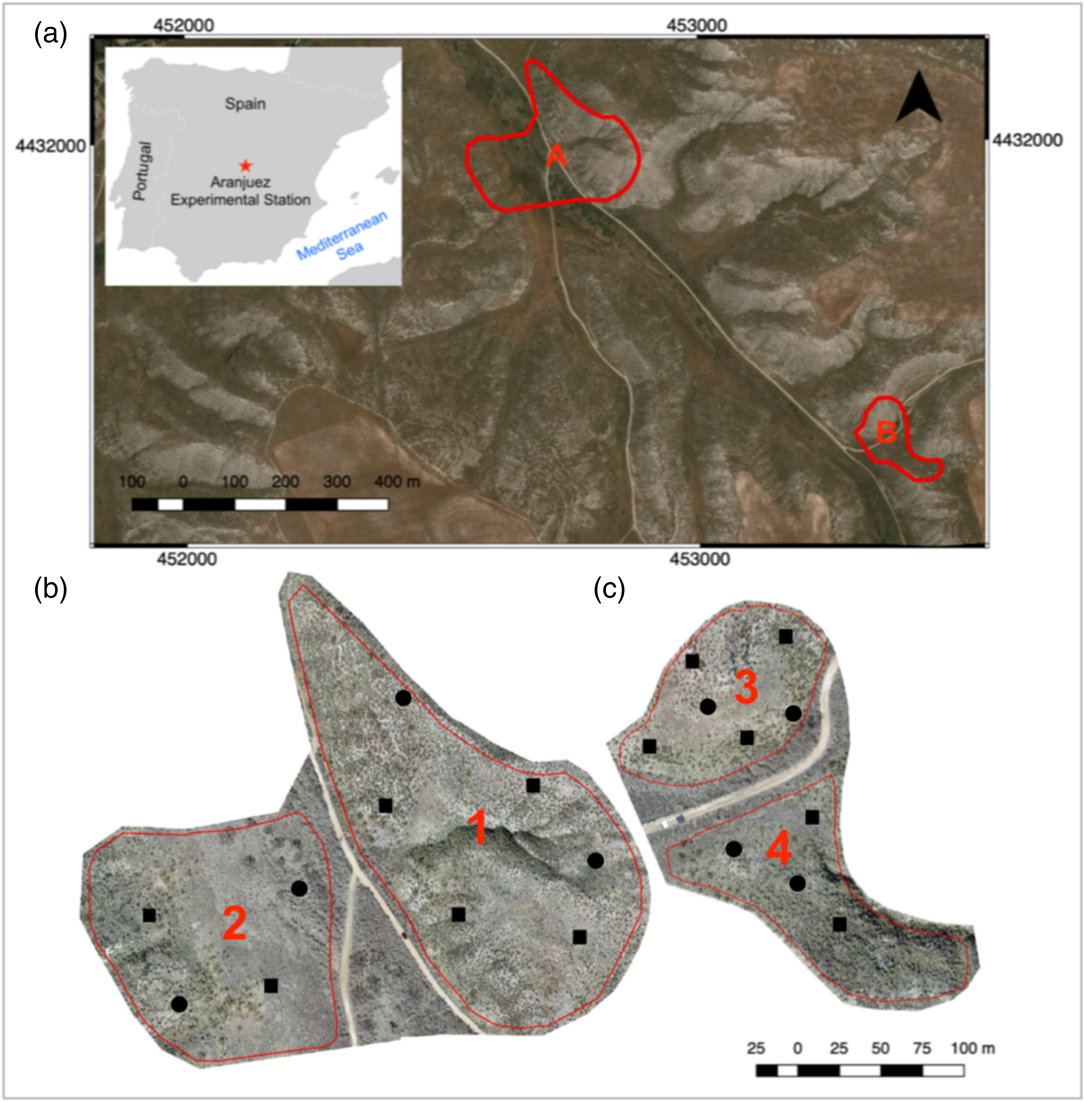
(a) Location of the study area in Aranjuez, central Spain; (b) study Area A and Zones 1 and 2; (c) study Area B and Zones 3 and 4. Black squares represent the ground control points (GCPs) used for the generation of the digital terrain model (DTM) and the orthomosaics and black circles are the ground validation points (GVPs) used to carry out an independent validation of the generated models [Colour figure can be viewed at wileyonlinelibrary.com]

This study was conducted in a typical dryland ecosystem at the Aranjuez Experimental Station, located in central Spain (40°020 N, 3°320 W; 590 m above sea level, Figure 1) (Ladrón de Guevara et al., 2018). The climate in this area is semi‐arid Mediterranean, with a mean annual temperature of 15°C and mean annual rainfall of 349 mm. The soil is classified as Gypsiric Leptosol (IUSS Working Group WRB, 2006). The vascular vegetation cover in this area is < 40% and is dominated by *Macrochloa tenacissima* tussocks and, in a lesser amount, small shrubs such as *Helianthemum squamatum* and *Gypsophila struthium*. On the surface area that is not covered by vascular vegetation, a rich biocrust community dominated by lichens develops, including *Diploschistes diacapsis, Squamarina lentigera* and *Psora decipiens* among others. A moss‐dominated crust also develops, with species such as *Pleurochaete squarrosa* and *Tortula revolvens*. Cyanobacteria genera, such as *Microcoleus, Tolypothrix* and *Nostoc* can also be found in the area (Cano‐Díaz et al., 2018). Refer to Maestre et al. (2013) for a complete list of species of the visible biocrusts in the study area. We worked in two different areas within the study site (Figure 1b): Area A, which covers an extension of 5.4 ha and is further sub‐divided in two zones (Zones 1 and 2). Zone 1 with a moderate slope and a dominant west exposure and Zone 2 with a smaller slope and a dominant east exposure. And Area B, which covers 1.2 ha and is also sub‐divided in two zones (Zones 3 and 4). Zone 3 presents an abrupt hill, mainly exposed to the south and Zone 4 a minor slope exposed to the north.

### 2.2 Field campaign

**FIGURE 2:**
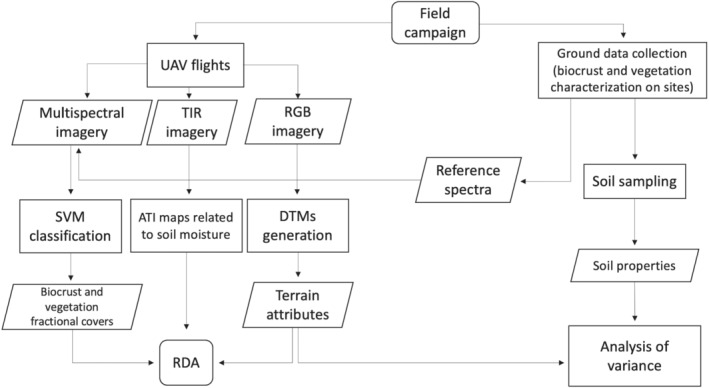
Workflow with the main processes carried out in this study. UAV, unmanned aerial vehicle; TIR, thermal infrared; RGB, red, green and blue; SVM, support vector machine; ATI, apparent thermal inertia; DTM, digital terrain model; RDA, redundancy analysis

In March 2018 a field campaign took place to characterize the biocrust, vascular vegetation cover and soils. We collected soil moisture samples and characterized the biocrust and vascular vegetation of the study area as described in Section 2.2.1. RGB, multispectral and TIR UAV‐based imagery was concurrently acquired to map the study area, derive terrain attributes and evaluate soil moisture. A general workflow with the main actions of this study is represented in Figure 2.

#### 2.2.1 Biocrust characterization and soil sampling

On 21 March 2018, we established 23 2 × 2 m^2^ observational field plots within the two areas. Within each plot the relative abundance of each type of surface component (biocrust, bare soil and vegetation) was visually assessed and the species composition of biocrust and vascular vegetation was characterized. Pictures were also taken with a camera mounted on a tripod at 1 m height. Three representative soil sub‐samples were taken in each plot from 0 to 10 cm depth (after removing the litter layer) and pooled to one sample, which represented the average soil characteristics of the plot. The samples were chemically and physically analysed in the laboratory applying standard techniques (Colombo & Miano, 2015). The samples were dried and sieved (2 mm mesh) and analysed to determine the percentage of soluble salts (SolSal), total phosphorus (P_tot_), percentage of organic carbon (OrgCarb) and total nitrogen (N_tot_). Soil moisture was measured in the field at 0–10 cm depth at three representative locations within each plot using a FieldScout TDR‐100 soil moisture meter (Spectrum Technologies, Fort Worth, TX, USA).

#### 2.2.2 Unmanned aerial vehicle (UAV) flights

The UAV flights (Table 1) were conducted using an eBee SQ (senseFly, Cheseaux‐sur‐Lausanne, Switzerland) fixed‐wing UAV operated by the company 3D Scanner (Zaragoza, Spain), on 22 March 2018 under clear‐sky conditions. These flights were conducted at the end of the rainy season in the study area because biocrusts are more colourful when are well hydrated by the rain. When colourful they are easier to map and differentiate using spectral information. In Supporting Information Figure S1, the daily precipitation as well as the minimum and maximum temperatures registered in the study area during 2018 are reported. Three different spectral cameras, i.e., S.O.D.A (senseFly), Parrot Sequoia (Parrot SA, Paris, France) and thermoMAP (senseFly) were flown to acquire RGB, multispectral and TIR images, respectively. Two sets of TIR imagery were acquired: the first before the sunrise, and the second at noon. During this second flight multispectral imagery was simultaneously acquired. A third flight, conducted immediately after the previous one, was performed to acquire RGB images on the site.

**TABLE 1:** Technical information of the unmanned aerial vehicle (UAV) flights using the thermoMAP, Parrot Sequoia and S.O.D.A. sensors over the Areas A and B

Flight	Sensor	Area flown	Start time (UTC)	Approximate duration (min)	Number of images collected	Average altitude above ground level (m)	Average ground sampling distance (cm)
1	thermoMAP	A	06:13	13	2397	69	13.09
2	thermoMAP	B	06:28	5	660	84	15.00
3	Sequoia	A	11:45	19	297	82	7.74
4	Sequoia	B	12:06	9	115	90	8.48
5	thermoMAP	A	11:52	13	2211	69	13.09
6	thermoMAP	B	12:06	5	597	84	15.00
7	S.O.D.A.	A	13:17	19	361	66	1.55
8	S.O.D.A.	B	13:06	9	135	76	1.79

All flights followed a single grid flight geometry at constant height, with flight lines parallel to the slope and with the cameras in nadir‐view position. Flights were done at average flight speeds between 10 and 12 m/s. Lateral and longitudinal overlaps of at least 75% and 70%, respectively, were achieved between the RGB images, with an average distance of 22 m between the flight lines and 20 m between images. Lateral and longitudinal overlaps of at least 85% and 70%, respectively, were obtained between the multispectral images, with a distance of 12 m between the flight lines and 24 m between images. Lateral and longitudinal overlaps of at least 80% and 95%, respectively, were achieved between the TIR images, with a distance of 16 m between the flight lines and 3 m between images. The multispectral Parrot Sequoia camera is a compact bundle of four cameras with complementary metal‐oxide‐semiconductor sensors, each one equipped with an individual narrow‐band filter (Assmann et al., 2018). This results in a multi‐band image composed of four bands: Green (550 ± 40 nm), Red (660 ± 40 nm), Red Edge (735 ± 10 nm) and NIR (790 ± 10 nm). The Red Edge band is located in the spectral region characterized by the rapid change in vegetation reflectance between red absorption due to pigment content and NIR reflectance due to the leaf structure. A white reference Micasense^®^ panel was measured with the Sequoia camera immediately before and directly after the drone survey to calibrate the images.

#### 2.2.3 Ground data

Reflectance factors were collected in the field to calibrate and validate the spectral data acquired from the UAV sensors using an ASD FieldSpec^®^ FR3 field spectroradiometer (Analytical Spectral Devices, Longmont, CO, USA) which features a spectral range from 350 nm to 2500 nm, an optical resolution of 1 nm and a nominal field of view of 25°. Two types of reference targets were measured during the flight campaign: white and black 2 × 2 m^2^ targets made of polyvinyl chloride (PVC)‐coated canvas material (Odyssey trademark material; Kayospruce Ltd, Fareham, UK) assumed to be lambertian surfaces, and selected homogeneous plots from the main biocrust communities in the study site. These measurements covered homogeneous surfaces of at least 30 cm in diameter. Before each target measurement, the downwelling solar radiance was measured on a calibrated 99% reflective Spectralon® panel (Labsphere Inc., Sutton, NH, USA) to optimize the instrument parameters and calculate the reflectance factors. Eight to ten spectra were taken on each target and were averaged to obtain the final spectrum for each target. These ground spectral measurements were acquired around solar noon, contemporaneously to the multispectral images collected by the Parrot Sequoia camera (UAV flight 2). Their exact location was registered using a differential global navigation satellite system (dGNSS) including one Topcon HiPer Pro antennas and one Topcon GPR‐1 (Topcon, Tokyo, Japan). The spectra of all ground measurements were resampled to the Sequoia spectral resolution using the response function of the sensor (Fawcett et al., 2020). These spectra were used to apply an empirical line correction (Smith & Milton, 1999) to the multispectral images to guarantee the radiometric quality in the final reflectance orthomosaics.

Simultaneous to the drone flights, the coordinates of 10 reference targets were taken in each area using the dGNSS. The targets were deployed in clear areas as black and white grids printed on thick paper sheets (297 mm × 420 mm). To optimize the geometric accuracy of the UAV‐derived products in both planimetry (*XY* direction) and altimetry (*Z* direction), the targets were evenly distributed across the whole study area of each zone, since the target distribution might affect later model generation accuracy (Gindraux et al., 2017; Martínez‐Carricondo et al., 2018) (Figure 1). In each area, six of the targets were used as ground control points (GCPs) for the generation of the orthophoto and DTM, while the remaining four were used as ground validation points (GVPs). Giving that the size of Area A and Area B were around 5.4 ha and 1.2 ha, respectively, the density of targets in the study site (111 and 600 GCP/km^2^ for Areas A and B, respectively) was enough to guarantee high accuracy in the derived digital models (Gindraux et al., 2017; Martínez‐Carricondo et al., 2018). We used a rapid‐static measurement technique and the coordinates were corrected during post‐processing (Hofmann‐Wellenhof et al., 2007). One of the two GNSS devices was used as master and the other as rover. The master station was placed in a clear flat area for a cumulative time of 5 h and was used as a static reference point. The rover was used to measure the centre point of each GCP and GVP, with the rover keept over the point for at least 2 min, taking a measurement every second with at least six satellites in the field of view of the receiver, according to the protocol proposed by Hegarty and Chatre (2008). The master and rover data were post‐processed using Topcon Tools software (Topcon Positioning Systems, Inc.). The dGNSS system used during the surveys has a horizontal precision of 3 mm + 0.5 ppm and a vertical precision of 5 mm + 0.5 ppm according to the Topcon HiPer Pro specification data.

### 2.3 Unmanned aerial vehicle (UAV) data processing

#### 2.3.1 Red, green and blue (RGB) images: Orthophoto, digital terrain model (DTM) and terrain attributes

RGB images acquired over Areas A and B of the study site by the S.O.D.A. camera were processed into orthophotos and grid‐based DSMs following a SfM workflow (Lucieer et al., 2014; Westoby et al., 2012) in Agisoft PhotoScan v.1.4 (Agisoft, St. Petersburg, Russia). A detailed description of the SfM algorithms used in Agisoft PhotoScan can be found in Verhoeven (2011). The images captured during the flights were aligned using an image feature recognition algorithm similar to Lowe's (2004) scale‐invariant feature transform method, which automatically detects and matches unique image feature characteristics that are stable under variations in view perspective and illumination across input photographs. This alignment results in a sparse three‐dimensional (3D) point cloud, which is used to create a dense point cloud by an iterative bundle adjustment algorithm. In turn this recreates its 3D geometry and camera positions from a sequence of two‐dimensional images acquired from multiple viewpoints (Ullman, 1983). The onboard navigation sensors allowed the camera positions and 3D point cloud to be automatically georeferenced within the precision of the instrument. The GCPs were then manually identified on the images, and their dGNSS coordinates were imported to optimize the spatial accuracy and improve the geometry of the 3D point cloud. A multi‐view stereo image matching algorithm was applied to the point clouds to increase the density of the point cloud and to convert them into the orthomosaics of each area. The GVPs were also identified in the images and they were only used for the model accuracy assessment in post‐processing and not for their creation. The geometric accuracy of the models was assessed by calculating the root mean square error (RMSE), the mean error and standard deviation of error of the GCPs and the GVPs.

**FIGURE 3:**
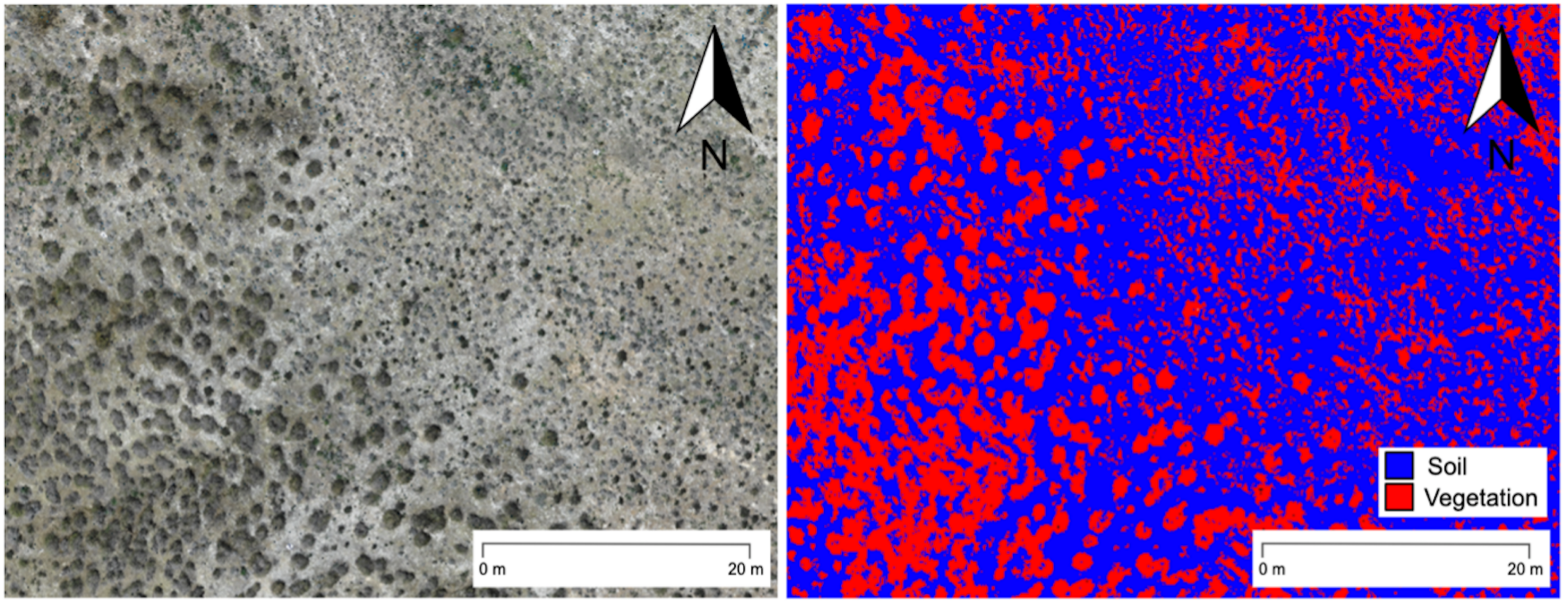
Example of vegetation classification derived from the point cloud used to derive the vegetation free digital terrain model (DTM) in Area A of the study site. Left: Red, green and blue (RGB) true colour composite with vegetation and bare soil areas. Right: Classification of vegetation (red) and bare soil (blue) [Colour figure can be viewed at wileyonlinelibrary.com]

To generate free‐of‐vegetation DTMs, the points belonging to vegetation were removed from the point cloud (Figure 3) using the CANUPO plug‐in (Brodu & Lague, 2012) in CloudCompare. For each area, two‐point clouds representing each class (i.e., vegetation and bare soil) were manually segmented from the point cloud. These sub‐clouds were used for training the classifier, computing a dimensionality descriptor on the original point cloud with a regular ramp of scale values (i.e., sampled from 0.1 to 1 with steps of 0.1). The classifier was applied to the point cloud (using selected core points) classifying and removing vegetation points. Finally, isolated points were removed by using the noise filter tool (i.e., specifying a radius of 0.3 m and a relative error) and the DTMs were exported from CloudCompare with a resolution of 10 cm, interpolating empty cells with average values. Free‐of‐vegetation DTMs were smoothed in ArcMap by applying an average filter with the Focal statistics tool by considering a circular window of 1 m diameter. This filter was applied to minimize potential errors that can occur with the removing vegetation procedure.

Besides elevation, the slope of each cell was also calculated from the filtered DTM, applying the nine parameter second‐order polynomial method (Zevenbergen & Thorne, 1987) within the *Slope* function in QGIS v.2.18.12. The aspect was derived using the *Aspect* function in QGIS v.2.18.12. The Northernness (i.e. the exposure of each cell of the DTM to the North) was calculated as the cosine of each cell of the aspect model. Since this variable ranges from −1 to 1, +1 was added to each pixel value to avoid problems in later calculations.

The contributing catchment area (CCA) of each pixel of the DTMs was modelled using the TauDEM toolbox (Tarboton, 1997; Tesfa et al., 2011) in ArcMap v.10.5. For this, the pits were first removed from the DTM and the *D‐Infinity Contributing Area* function was used to calculate it. The D‐Infinity algorithm (Tarboton, 1997) calculates the flow direction using the steepest downwards slope on the eight triangular facets formed in a 3 × 3 pixel window centred on the grid cell of interest. The topographic wetness index (TWI; Beven & Kirkby, 1979), which quantifies the topographic control on hydrological processes, was calculated for each cell according to Equation 1:

(1) $$\mathrm{TWI}=\ln\left(\frac{A}{\tan\beta }\right)$$

where *A* is the upstream area for each pixel and *β* is the slope in degrees.

The length slope factor (LSF; Renard et al., 1997), an indicator of the potential sediment transport or erosion risk under specific slope conditions, was calculated according to Equation 2:

(2) $$\mathrm{LSF}=\left(n+1\right)\times {\left(\frac{A}{22.13}\right)}^{n}\times {\left(\frac{\sin\beta }{0.0896}\right)}^{m}$$

where LSF is the length slope factor, *A* the CCA, *β* the slope gradient and *n* and *m* are constant parameters set to 0.4 and 1.3, respectively.

The potential solar incoming radiation (PSIR) was calculated using the vegetation filtered DTMs of each area and the geometric solar radiation model implemented in ArcGIS Solar Analyst tool by assuming a uniform clear sky condition with a constant transmissivity of 0.5 (Fu & Rich, 1999) and a diffuse radiation proportion of 0.3. The map of the whole year PSIR (Wh/m^2^/yr) was used in this study as an indicator of the potential evapotranspiration (Monteith & Szeicz, 1962).

#### 2.3.2 Multispectral Parrot Sequoia images

The multispectral images were processed in Pix4DMapper (Pix4D S.A., Lausanne, Switzerland) to obtain the final reflectance for each spectral band of the image. First, the calibration coefficient *K* for converting measured pseudo‐radiance *R* to reflectance was derived for each band as in Equation 3:

(3) $$K=\frac{\rho_{\mathrm{ref}}}{R_{\mathrm{ref}}}$$

where *ρ*_ref_ is the known reflectance of the panel for the Sequoia band and *R*_ref_ represents the measured pseudo‐radiance averaged over the pixels of the reference panel. Values for *ρ*_ref_ were provided by the manufacturer. The digital number (*P*) of each pixel were converted to pseudo‐radiance in arbitrary units *R* according to Equation 4:

(4) $$R=f^{2}\frac{P‐B}{A\gamma \varepsilon +C}$$

where *γ* is the ISO number, *ε* is the exposure time in seconds, *f* is the *f*‐number and *A*, *B* and *C* are camera‐specific calibration coefficients which model the non‐linear behaviour of the sensor (Fawcett et al., 2020).

Finally, the reflectance per pixel (*ρ*) of the scene was derived as in Equation 5:

(5) ρ=KR

To correct the reflectance measured by the multispectral sensor, the empirical line method (Smith & Milton, 1999) was applied. This method was based on an empirical relationship between the drone reflectance and ground reflectance of the reference targets (i.e., black and white reference panels) collected with the ASD Field Spectroradiometer in the field.

The white panel saturated on the Sequoia images, therefore homogeneous biocrust targets were used as bright targets instead. The linear equation obtained for each multispectral band was applied to obtain the final reflectance of the multispectral images.

An orthomosaic was built from the multispectral images covering the study area with an original spatial resolution of 7.74 cm and 8.48 cm in Areas A and B, respectively, further resampled to 10 cm/pixel. The geometric accuracy of the orthomosaic was assessed by calculating the RMSE, the mean error and standard deviation error of the GCPs and the GVPs.

The CR algorithm was computed on the geometrically and radiometrically corrected multispectral orthomosaic. This method normalizes the reflectance spectrum to a common baseline at specified wavelengths and allows the comparison of absorption features in the spectra that are produced by target components, for example pigments for vegetation covers. This is achieved by approximating the continuum between local spectral maxima through straight‐line segments; a value of 1 is assigned to the local maxima, and a value between 0 and 1 is obtained in correspondence of the absorption features. Interpolating the reflectance between 550 nm (Green band) and 735 nm (Red Edge band) as the continuum baseline. Although this algorithm is more typically applied to hyperspectral data, it has already been used in multispectral imagery to map drylands (Panigada et al., 2019) and vegetation parameters (Chauhan et al., 2020). The absorption feature related to chlorophyll‐*a* was computed at 660 nm by extracting the value of the CR in the red band (CR_red_). The CR_red_ was used to improve later classification of the images.

**FIGURE 4:**
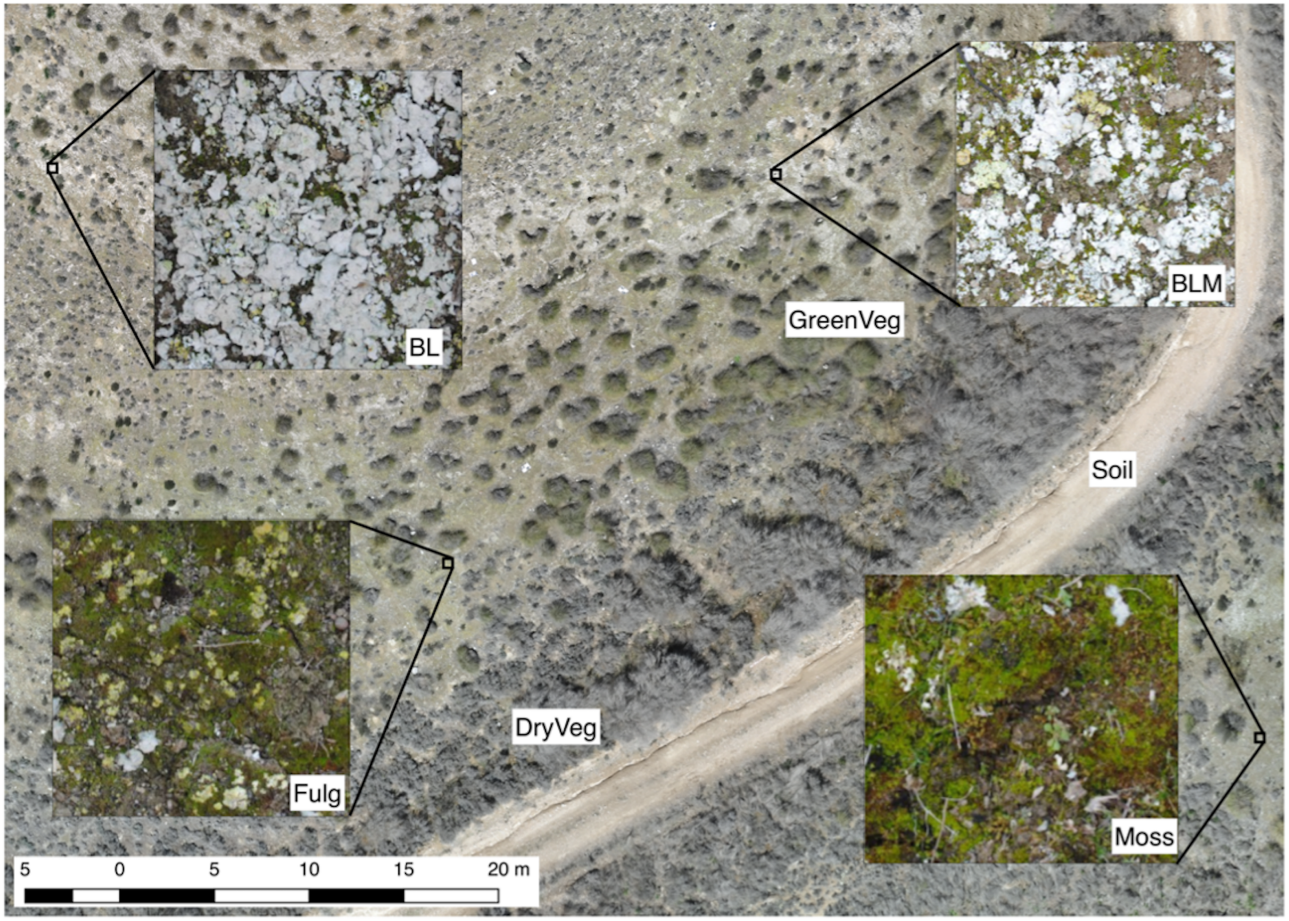
Orthomosaic of Area B and details of the classes used to classify the multispectral images. BL, bright lichens; BLM, bright lichens and moss; Fulg, *Fulgensia* spp. and moss; GreenVeg, green vegetation; DryVeg, dry vegetation [Colour figure can be viewed at wileyonlinelibrary.com]

Seven different surface covers were identified in the study site during the field surveys (Figure 4): bare soil (Soil), bright lichens (BL; patches of *Squamarina* spp., *Diploschistes* spp., *Buellia* spp.), bright lichens with moss (BLM; *Squamarina* spp., *Diploschistes* spp., *Buellia* spp. and *Tortula revolve*ns [Zone 1] or *Syntrichia ruralis* [Zone 2]), moss (Moss; *Tortula revolvens* [Zone 1] and *Syntrichia ruralis* [Zone 2]), *Fulgensia* spp. and moss (Fulg; *Fulgensia* spp. and *Tortula revolvens* [Zone 3] or *Pleurochaete squarrosa* [Zone 4]), green vegetation (GreenVeg; green tussocks of *Macrochloa tenacissima*) and dry vegetation (DryVeg; dry individuals of *Helianthemum squamatum*, *Gypsophila struthium* and *Macrochloa tenacissima*). These surface covers were used as reference for the digital classification of the multispectral orthomosaic.

At least 15 polygons (around 100 pixels) per class were selected on each orthomosaic to train the support vector machine (SVM) algorithm used in the classification. These training areas were selected using ancillary information such as field notes, global positioning system (GPS) points and high resolution RGB orthomosaics. The four bands of the multispectral images were used as input in the SVM.

SVM is a statistical learning based supervised classification method (Cortes & Vapnik, 1995; Vapnik, 2005). It uses training samples of the target categories and separates them using a decision surface called the ‘hyperplane’, which maximizes the margin between each category. The support vectors are the closest training samples to this hyperplane, and are the ones used by the algorithm. In this study, a radial basis kernel function was used. The SVM was used to classify the images and derive thematic maps with the spatial distribution of the main land covers in the two areas. CR_red_ was used to improve the discrimination of some categories with similar spectral behaviour such as DryVeg and Moss, but different pigment absorption. Pixels classified as DryVeg with CR_red_ values lower than 0.75 were assigned to Moss and pixels of BL with CR_red_ values of 1 were assigned to Soil. These thresholds were defined from the CR_red_ mean and standard deviation values calculated in correspondence with the training polygons of soil, DryVeg and Moss classes.

The classification accuracy was assessed through confusion matrices (Story & Congalton, 1986). In the confusion matrix the ground truth data (i.e., columns) are compared to the classified data (i.e., rows). The major diagonal represents the agreement between these two data sets, and the overall accuracy (OA) of the classification is calculated by dividing the sum of the entries of this diagonal by the total number of samples taken. In addition, the Kappa coefficient (*κ*, Cohen, 1960) was calculated to asses the classification accuracy. The accuracy of the classification over Area A and Area B was assessed with two validation datasets, each consisting in 10 polygons of nine pixels for each class (90 pixels per class). Polygons were manually defined using ancillary information such as field pictures, field notes, GPS points and high‐resolution RGB orthomosaics.

#### 2.3.3 Thermal imagery and apparent thermal inertia (ATI) calculation

TIR images acquired by the thermoMAP camera were calibrated during the flight using an integrated shutter for in‐flight radiometric calibration. This shutter automatically closes after every picture is captured and self‐calibrates by comparing the grey level of each photograph with the temperature measured by the built‐in temperature sensor of the camera. The raw images were converted using Pix4D software (Pix4D) to temperature in Celsius degrees according to Equation 6:

(6) T=0.01R−100

where *T* is the absolute temperature in Celsius degrees and *R* is the radiometric value of thermoMap thermal images. The emissivity of field sampled biocrusts and soils was measured in the laboratory and found to be close to 1 for both targets (see Supporting Information, Table S1) in the range of the thermoMAP (8.5–13.5 μm). Since the aim of using these images in this study was to measure properties related to biocrusts, emissivity was therefore neglected and thus not included in this calculation.

Two orthomosaics (one captured before sunrise and one at noon) of each target area with a spatial resolution of 15 cm were generated using Pix4Dmapper (Pix4D). The geometric accuracy of these TIR orthomosaics was assessed by calculating the RMSE, the mean error and standard deviation of error of the GCPs and the GVPs.

Land surface temperature differences and albedo were used in the definition of the concept of apparent thermal inertia to assess the space variability of soil water content (Negm et al., 2017; Verstraeten et al., 2006). We applied the apparent thermal inertia (ATI; in K^−1^), which is an approximation of thermal inertia and is derived directly from multi‐spectral remote sensing imagery (Mitra & Majumdar, 2004; Van Doninck et al., 2011) according to Equation 7:

(7) $$\mathrm{ATI}=C\frac{1-\alpha }{∆T}\;$$

where ∆*T* is the amplitude of the diurnal temperature range calculated as the difference between the maximum and the minimum daily surface temperature, *α* is the surface spectral albedo and *C* is the solar correction factor that changes over space and time to normalize for solar flux variations with latitude and solar declination changes between seasons. The ∆*T* was calculated as the difference between the surface temperature of the orthomosaic captured at noon and the one captured before the sunrise. The value of *C* was calculated and had a value of 1.19. We approximated the albedo as in Equation 8:

(8) $$\alpha =\frac{\rho \text{GREEN}+\rho \mathrm{RED}+\rho \mathrm{REG}+\rho \mathrm{NIR}}{4}$$

where *ρ*GREEN, *ρ*RED, *ρ*REG and *ρ*NIR are the reflectance values in the Green, Red, Red Edge and NIR bands, respectively. Multispectral bands were calibrated so that the brightness map can reasonably be considered to be a proxy of the broadband visible albedo. The correlation between thermal inertia and soil moisture, observed in the literature (Minacapilli et al., 2009), was here tested through a linear regression model between the mean ATI value extracted from orthomosaics and the mean soil moisture measured on each plot during the field campaign by traditional TDR methods. The average value of ATI on each 2 × 2 m^2^ plot was obtained in QGIS v.2.18.12 by averaging the ATI values of the corresponding polygons.

Vegetation pixels were excluded from our analysis. The high‐spatial resolution of TIR and multispectral images allowed us to use the classification of the multispectral images to create a vegetation‐free mask that was applied to the ATI orthomosaics.

### 2.4 Statistical analysis

To evaluate if there were statistically significant differences between the four study zones, a *post hoc* Dunn's test followed by a Kruskal–Wallis test was performed, using the *dunnTest* function from the FSA package (Ogle et al., 2021) in R. To analyse the effect of the terrain attributes in relation to the spatial distribution of soil properties measured in the sampling plots, the correlation ratio (Pearson, 1926) was calculated between the terrain attributes obtained from the RGB images and the ATI and the soil characteristics retrieved from ground measurements. The average value of the terrain attributes on each plot was obtained in QGIS v.2.18.12 by averaging the terrain attribute values of the corresponding polygons, excluding vegetation pixels. The correlation ratio was calculated as in Equation 9. It can assume values in the interval (0,1), and gives an indication of how much the data variance (in our case the soil property) is explained by the terrain factors; a high correlation ratio (close to 1) means that the factor explains most of the data variance. The weighted variance of the category means divided by the variance of all the samples. In our case the values of each terrain attribute (category) were divided in three classes standardized using their maximum and minimum values (i.e. low, medium, high):

(9) $$\eta^{2}=\frac{\sigma_{\underline{y}}^{2}}{\sigma_{y}^{2}},\;\text{where}\;\sigma_{\underline{y}}^{2}=\frac{\sum_{x}n_{x}{\left({\underline{y}}_{x}-\underline{y}\right)}^{2}}{\sum_{x}n_{x}}\;\text{and}\;\sigma_{y}^{2}=\frac{\sum_{x,i}n_{x}{\left(y_{\mathrm{xi}}-\underline{y}\right)}^{2}}{n}$$

where *y*_*xi*_ is the single soil property observation, *x* indicates the terrain attribute category, and *i* indicates an observation. If *n*_*x*_ is the number of observations in the *x* terrain category, ${\underline{y}}_{x}$ is the mean of the category *x* and $\underline{y}$ is the mean of the whole population.

The relationship between the different biocrust covers and the terrain variables in the whole study area was evaluated through a redundancy analysis (RDA) performed using the *vegan* R package (Oksanen et al., 2017) v.2.4. To test the significance of the selected variables in the RDA, permutation tests (*N* = 999) were performed using the marginal effect of the terms in the *anova*.*cca* function.

The CCA variable was removed from the analysis since it was used to calculate TWI and LSF. Based on this, a set of seven terrain attributes were considered in the analysis: (i) PSIR as an indicator of the potential evapotranspiration (Monteith & Szeicz, 1962) and as a proxy of thermal microclimate (Durham et al., 2018; Suggitt et al., 2018); (ii) LSF as an index of surface stability (Renard et al., 1997); (iii) TWI as an index of the topographic control on hydrological processes (Sörensen et al., 2006); (iv) elevation; (v) Slope gradient, as it has implications for dryland components distribution not captured by other variables (Rodríguez‐Caballero et al., 2019); (vi) Northernness, as indicator of facing of the slope and (vii) ATI, as a proxy of soil moisture. The vegetation (combination of DryVeg and GreenVeg) and soil fractional covers were also included as variables in this analysis. The dataset used in this analysis was obtained through a random sampling of 1147 points in the study area, with a minimum distance of 3 m between them to avoid spatial autocorrelation. This distance was calculated through a semi‐variogram analysis (Curran, 1988). A circle of 3 m was drawn around each point to extract the terrain attributes and the fractional cover of the different components (i.e., soil, vegetation and different biocrust compositions). Several models with all combinations of variables were computed to identify the best‐fitting RDA model based on maximum adjusted *r* ^2^, but avoiding multicollinearity. This was estimated by the variance inflation factor (VIF), which was calculated through the *vif.cca* function of *vegan* R package. Variables with a VIF higher than 10 were excluded from the analysis.

## 3 RESULTS

### 3.1 Unmanned aerial vehicle (UAV) imagery geometric accuracy

The geometric accuracy of the products generated from the UAV imagery was assessed based on the RMSE, mean and standard deviation of the GCPs used for model generation and the GVPs used for the assessment of model accuracy. A summary of the statistics is reported in Table 2. The total RMSE computed on the GVPs was lower than 3.2 cm for both RGB models, about 22 cm for the multispectral images and lower than 25 cm for the TIR images. The mean error over the whole study area was lower than 1 cm in the RGB models. For the multispectral and the TIR images, the mean error was always lower than 2 cm, in many cases lower than 1 cm. Focusing on the RGB survey used to derive the DSMs, DTMs and terrain attributes, the mean vertical error and standard deviation were very low. This suggests that the generated models and terrain attribute maps were not affected by systematic error.

**TABLE 2:** Root mean square error (RMSE), mean error (ME) and standard deviation of error (STDEV) for red, green and blue (RGB), multispectral and thermal infrared (TIR) imagery and both ground control point (GCP) and ground validation point (GVP)

		GCP	GCP	GCP	GVP	GVP	GVP
Area	Imagery	RMSE *XY* (cm)	RMSE *Z* (cm)	Total RMSE (cm)	RMSE *XY* (cm)	RMSE *Z* (cm)	Total RMSE (cm)
A	RGB	2.17	4.2	4.7	2.2	2.3	3.2
B		1.58	1.3	2.1	1.9	1.3	2.4
A	Multispectral	18.8	21.8	22.4	19.4	13.7	21.8
B		11.1	17.1	18.2	13.7	21.4	22.3
A	TIR	21.2	23.4	24.7	23.1	22.4	24.5
B		16.6	17.3	19.8	16.8	15.9	19.9

		ME|STDEV *X* (cm)	ME|STDEV *Y* (cm)	ME|STDEV *Z* (cm)	ME|STDEV *X* (cm)	ME|STDEV *Y* (cm)	ME|STDEV *Z* (cm)
A	RGB	0.09| 0.49	0.02| 0.91	0.25| 1.40	0.09| 0.51	0.01| 0.49	0.17| 0.95
B		0.08| 0.16	0.01| 0.13	0.03| 0.41	−0.1| 0.41	0.01| 0.13	−0.04| 0.47
A	Multispectral	0.83| 4.28	0.15| 4.72	1.2| 6.7	−1.27| 8.45	−0.25| 2.89	−1.8| 2.27
B		−0.6| 1.1	0.2| 1.7	−0.3| 3.5	0.9| 2.6	−0.3| 2.5	0.4| 6.1
A	TIR	0.93| 4.82	0.16| 5.32	1.32| 5.62	1.01| 5.25	0.15| 5.09	1.31| 5.57
B		−0.89| 1.7	0.26| 1.77	−0.3| 2.03	−0.91| 1.72	0.24| 1.63	0.33| 2.04

### 3.2 Classification of the multispectral orthomosaics and evaluation of the classification accuracy

**FIGURE 5:**
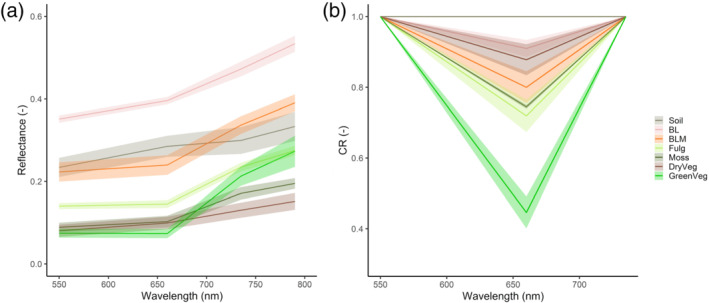
Mean reflectance and standard deviation spectra (a) and mean continuum removed (CR) and standard deviation spectra (b) of the vegetation, biocrust and soil classes used as training for the support vector machine (SVM) classification. DryVeg, dry vegetation; GreenVeg, green vegetation; BLM, bright lichens with moss; Fulg, *Fulgensia* spp. with moss [Colour figure can be viewed at wileyonlinelibrary.com]

Mean reflectances and standard deviations of the training classes used for the classification procedure are shown in Figure 5(a). Classes dominated by lichens had higher reflectance, with BL the highest reflectance class among them. Classes dominated by mosses had the lowest reflectance among biocrusts, only DryVeg reflectance was lower. The spectra CR shown in Figure 5(b) highlights the absorption due to chlorophyll at ~660 nm. Moss was the biocrust class with the highest absorption at ~660 nm due to chlorophyll (i.e., lowest CR_red_ value), while GreenVeg was the class showing the highest absorption of all the evaluated classes. While similar in the spectral shape, DryVeg and Moss presented differences in their CR_red_ and reflectance values. The red absorption feature was present in all classes but Soil, and allowed to better differentiate it from biocrust classes. These subtle differences allowed an improvement in the classification carried out by the SVM, which slightly confused these two classes, by using thresholds in the CR_red_.

**FIGURE 6:**
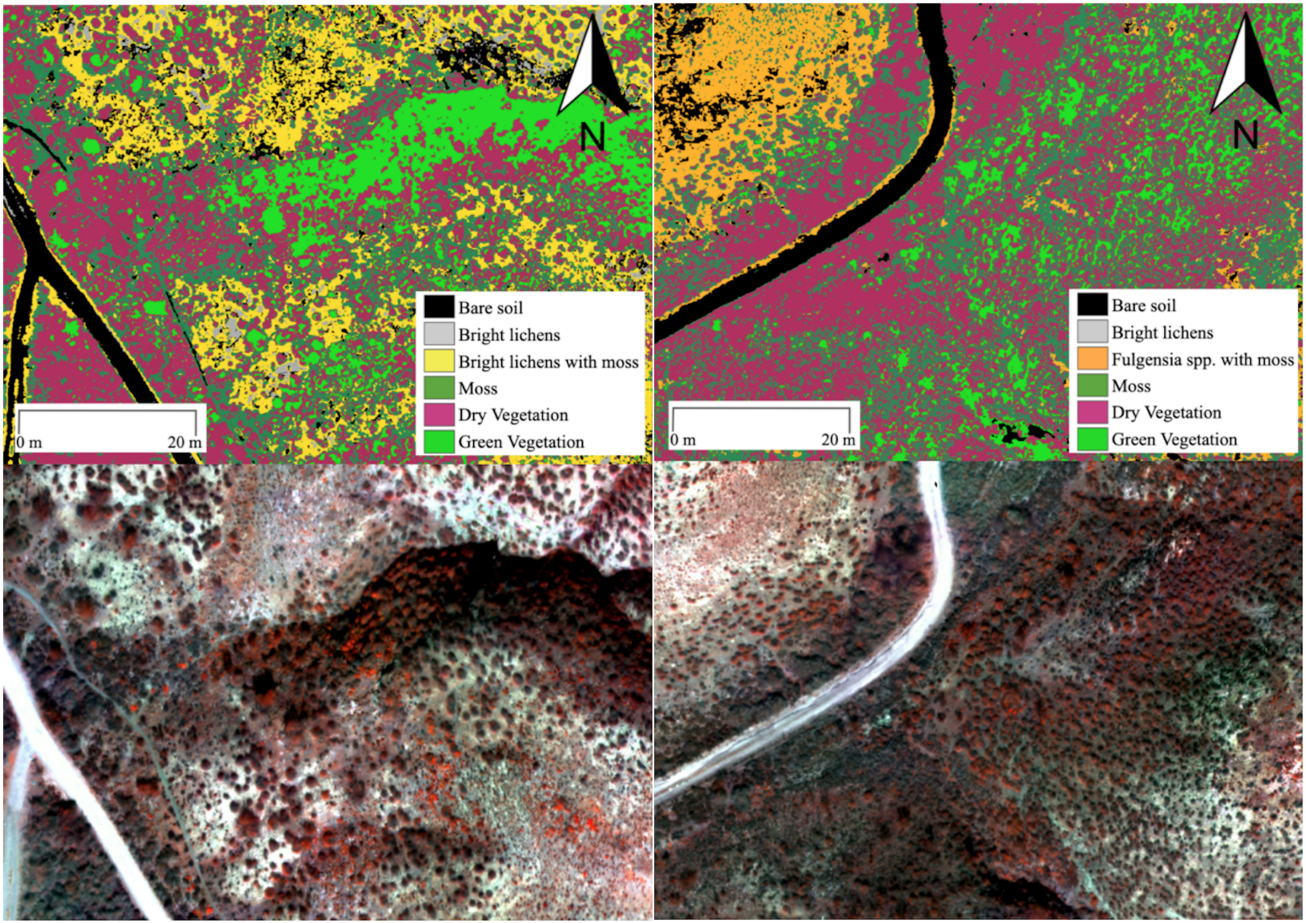
Support vector machine classifications (top) of the multispectral images (bottom), represented as false‐colour composite (near infrared [NIR], green and red bands), in the six principal surface components. Bare soil, patches of bare soil; Bright lichens, patches of *Squamarina* spp., *Diploschistes* spp., *Buellia* spp.; Bright lichens with moss, patches of *Squamarina* spp., *Diploschistes* spp., *Buellia* spp. and moss; Moss, *Tortula revolvens*, and/or *Syntrichia ruralis* and/or *Pleurochaete squarrosa* ; Fulg, *Fulgensia* spp. and moss; Dry vegetation, dry individuals of *Helianthemum squamatum*, *Gypsophila struthium* and *Macrochloa tenacissima* ; Green vegetation, green tussocks of *Macrochloa tenacissima* . Left: study Area A; right: study Area B [Colour figure can be viewed at wileyonlinelibrary.com]

The SVM classification generated land cover maps of the two target areas in the study site (Figure 6). The SVM classifications were improved using the CR_red_ thresholds on the Soil and DryVeg classes. Final accuracies are reported in Table S2, OAs higher than 80% in most cases were achieved in both areas, with *κ* equal to 0.8 and 0.93 in areas A and B, respectively. Only BLM in Area A had an OA below 80%. As shown in the confusion matrices (Table S2), BL and GreenVeg were the most accurately classified covers in this area, while there was 10% of Moss misclassified as BLM. There was some confusion between Moss and DryVeg, with 10% of Moss misclassified as DryVeg in Area A, and between BLM and Soil, with 14% of BLM classified as Soil. In both areas, the most dominant class was DryVeg, while the least ones were the classes with lichens and mosses mixed (i.e., BLM and Fulg in Area A and Area B, respectively; Table 3).

**TABLE 3:** Fractional cover of each class in the study areas as resulted from the classification of the multispectral images

	Surface cover (%)						
	Soil	DryVeg	GreenVeg	BL	BLM	Fulg	Moss
Area A	4.01	59.88	10.23	1.32	10.21	—	14.32
Area B	8.94	43.11	8.03	0.86	—	14.84	24.19

*Note:* — indicates class not present. Soil, bare soil; DryVeg, dry vegetation; GreenVeg, green vegetation; BL, bright lichens; BLM, bright lichens and moss; Fulg, *Fulgensia* spp. and moss; Moss, moss.

### 3.3 Thermal data and soil moisture

**FIGURE 7:**
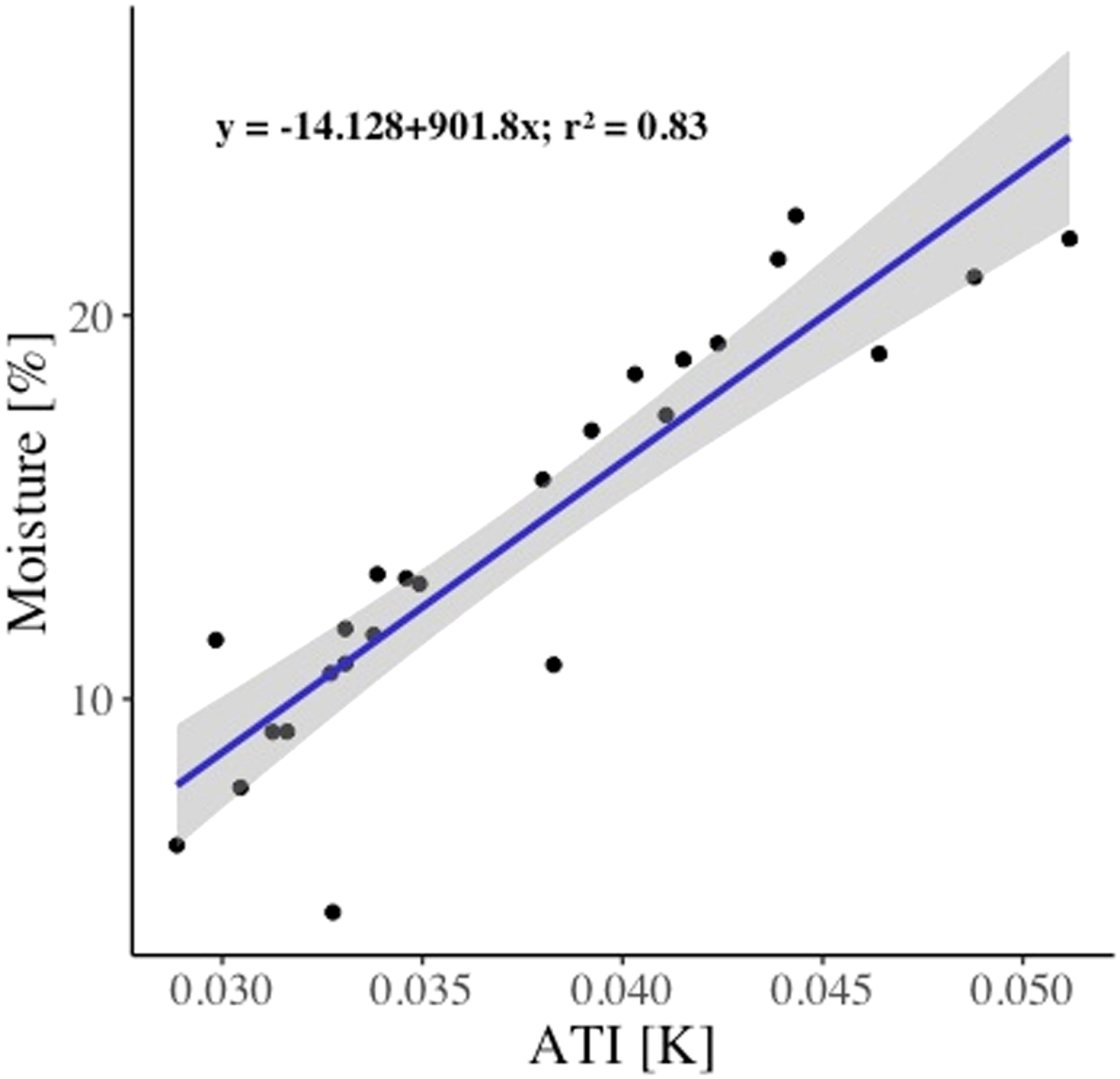
Linear regression between apparent thermal inertia (ATI) and soil moisture in the 23 field plots used in this study. Shaded areas represent ±95% symmetrical confidence interval [Colour figure can be viewed at wileyonlinelibrary.com]

The soil moisture values measured in the 23 field plots were similar to that of the winter long‐term soil moisture of this area (Lafuente et al., 2018), confirming that the moisture status we captured with the drone flight was representative of the winter rainy season. ATI values correlated well with the soil moisture (*r* ^2^ = 0.83; Figure 7), indicating that ATI is a good estimator of soil moisture, even when soils are covered by biocrusts. ATI was then used as a proxy of soil moisture for vegetation‐free areas in the subsequent analysis.

### 3.4 Analysis of soil properties

Soils in the study area are Gypsiric Leptosols and are characterized by the very high content of SalSol, reaching values higher than 90% in some of the plots, and the low presence of OrgCarb (Table 4). Soil samples showed similar low nutrient contents in all plots, reaching values of P_tot_ of 0% and values of N_tot_ close to 0% in some of them. Moisture presented high variability among the plots, with mean values around 14%.

**TABLE 4:** Soil attributes retrieved from the 23 2 × 2 m^2^ plots sampled in the field

Soil attribute	Mean	Maximum	Minimum	Standard deviation
Moisture (%)	14.3	22.6	4.44	5.3
SolSal (%)	81	96	19	15.02
OrgCarb (kg/m ^2^ )	0.92	3.96	0.14	0.74
P_tot_ (%)	0.6	7.26	0	1.44
N_tot_ (%)	0.08	0.37	0.01	0.07

*Note:* SolSal, soluble salts; OrgCarb, organic carbon; P_tot_, total phosphorus; N_tot_, total nitrogen.

### 3.5 Statistical analysis

#### 3.5.1 Relationship between terrain attributes and soils at plot level

**FIGURE 8:**
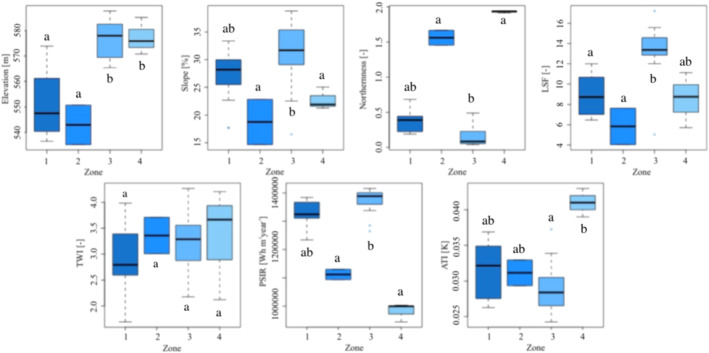
Box plots of the terrain attributes for the different aspects of the plots deployed in the field. The different lowercase letters indicate statistically significant differences between aspects (*post hoc* Dunn's test following a Kruskal–Wallis test). 1, 2, 3 and 4 indicate the zones of the study area, which are mainly exposed to west, east, south and north, respectively. LSF, length slope factor; TWI, topographic wetness index; PSIR, potential solar incoming radiation; ATI, apparent thermal inertia [Colour figure can be viewed at wileyonlinelibrary.com]

Terrain attributes in the field plots were found to vary across the four zones sampled (Figure 8). The elevation presented similar values in Zones 1 and 2 (Area A) and in Zones 3 and 4 (Area B). The slope was higher in Zone 3, reaching values of 35% of slope gradient. As expected, LSF presented similar patterns as slope. The TWI presented values around 3.5 with values not statistically different in the four zones. PSIR showed an opposite pattern compared to Northernness, with higher values in Zones 1 and 3, which are mainly exposed to southwest and south, respectively, indicating higher evapotranspiration. PSIR presented lower values in Zone 2 mainly exposed to the east and minimum values in Zone 4, mainly exposed to the north. Similar to Northernness, ATI, that is, soil moisture, presented the highest values in Zone 4 and lower values in Zone 1, evidencing that the soil moisture is related to the aspect.

Among the terrain attributes investigated, Northernness, ATI and PSIR are the ones that explained most of the variance of the soil properties (Table 5). Soils more exposed to north and characterized by higher ATI show a lower content of SolSal and higher content of OrgCarb, P_tot_ and N_tot._ Soils characterized by higher values of PSIR show an opposite trend, with higher content of SolSal and lower content of OrgCarb, P_tot_ and N_tot_. The variance of soil properties is similarly explained by Northernness, ATI and PSIR with values of *η* ^*2*^ close to 0.4 when related to SalSol, OrgCarb, P_tot_ and N_tot_, with slightly higher values of *η* ^*2*^ driven by PSIR.

**TABLE 5:** Descriptive statistics (i.e., mean and standard deviation) of soil properties grouped in terciles of terrain attributes

	L	M	H	*η* ^*2*^
*North*				
SolSal	86.50 ± 4.74	68.60 ± 4.94	62.50 ± 29.21	0.43
OrgCarb	1.24 ± 0.57	1.39 ± 0.62	3.27 ± 2.49	0.35
P_tot_	112.72 ± 30.51	167.01 ± 65.05	219.50 ± 151.14	0.31
N_tot_	0.06 ± 0.03	0.07 ± 0.05	0.17 ± 0.13	0.35
*ATI*				
SolSal	86.09 ± 3.80	83.30 ± 12.02	56.00 ± 32.04	0.42
OrgCarb	1.18 ± 0.49	1.19 ± 0.53	3.30 ± 3.04	0.32
P_tot_	112.81 ± 28.91	119.00 ± 48.69	251.66 ± 167.50	0.37
N_tot_	0.06 ± 0.02	0.06 ± 0.03	0.18 ± 0.15	0.35
*PSIR*				
SolSal	56.00 ± 30.04	78.25 ± 12.31	86.31 ± 4.67	0.45
OrgCarb	3.30 ± 3.04	1.28 ± 0.38	1.25 ± 0.61	0.35
P_tot_	251.66 ± 167.50	125.00 ± 68.01	116.43 ± 27.38	0.37
N_tot_	0.18 ± 0.15	0.06 ± 0.03	0.06 ± 0.03	0.36

*Note:* North, northernness; ATI, apparent thermal inertia, PSIR, potential solar incoming radiation. L, low; M, medium; H, high. Correlation ratio (*η* ^*2*^) among soil variables and topography variables is also reported. Only variables with *η* ^*2*^ higher than 0.3 are represented. SolSal, soluble salts (%); OrgCarb, organic carbon (km/m^2^); P_tot_, total phosphorus (%); N_tot_, total nitrogen (%)

#### 3.5.2 Relationships between topography, biocrusts and vegetation distribution at landscape level

**FIGURE 9:**
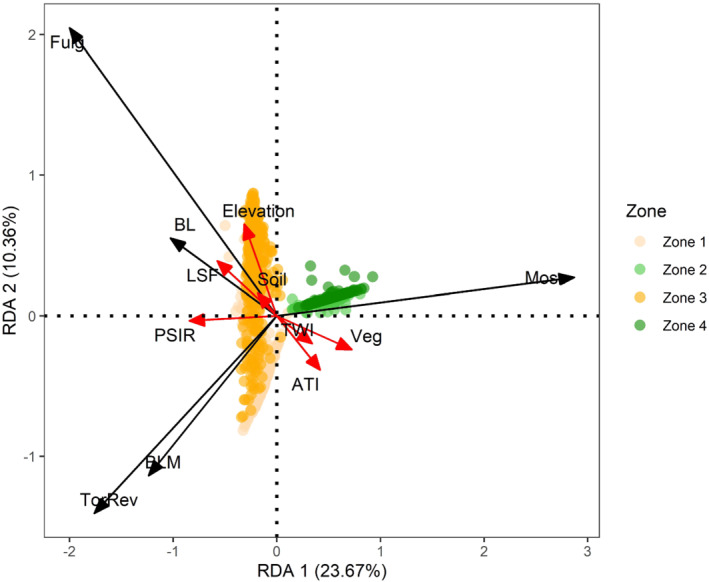
Redundancy analysis (RDA) of the biocrust and vegetation cover with respect to terrain attributes, soil and vegetation cover in the four zones of the study area. The red arrows indicate the increase along terrain attribute gradients, while the black arrows indicate the response variables (biocrust classes) and their directions represent the explanatory relationships with the axis. LSF, length slope factor; ATI, apparent thermal inertia; TWI, topographic wetness index; Veg, presence of vascular vegetation; Soil, presence of bare soil; PSIR, potential solar incoming radiation; BL, bright lichens; BLM, bright lichens with moss; Fulg, *Fulgensia* spp. with moss; TorRev, *Tortula revolvens*; Moss, *Pleurochaete squarrosa* and *Syntrichia ruralis* [Colour figure can be viewed at wileyonlinelibrary.com]

The best fitted RDA model excluded Northernness and Slope variables, which showed multicollinearity, with VIF values around 20. The RDA results indicated that terrain variables significantly explained variation in the biocrust distribution in our study area (39.8% of the total inertia; *F* = 92.5; *p* < 0.001). The first two RDA‐axes accounted for 34.0% of the variation for biocrust data (Figure 9). The most significant variables were: ATI (*F* = 115.2; *p* < 0.001), Elevation (*F* = 100.8; *p* < 0.001), Northernness (*F* = 32.0; *p* > 0.001) and Veg (*F* = 29.7; *p* < 0.001) (Table S3). For those variables, differences were found between areas dominated by lichens and mosses. Lichen‐dominated biocrusts appeared on the left side of the first axis, which was characterized by low values of vegetation presence and ATI (i.e., low moisture) and high values of LSF, which indicates high potential soil erosion, from a topographic point of view and high values of PSIR and Elevation. As evidenced in Table 5, low ATI and high PSIR values are related to high SolSal content and low OrgCarb, P_tot_ and N_tot._ Among lichen‐dominated biocrusts, Fulg class development was mostly driven by the gradient of elevation, BL was controlled by high LSF and BLM was driven by low ATI and low elevation. Conversely, the distribution of mosses typical of shaded areas (i.e., *Pleurochaete squarrosa* and *Syntrichia ruralis*) appeared on the left side of the first axis, where vegetation presence and soil moisture (high ATI values) were higher and soil was richer in OrgCarb, P_tot_ and N_tot_. Nevertheless, *Tortula revolvens* develops in soils with similar characteristics to those where lichens dominate, characterized by low elevation and low ATI and vegetation presence.

## 4 DISCUSSION

Previous studies have evaluated the effect of the terrain on biocrust spatial distribution (Durham et al., 2018; Rodríguez‐Caballero et al., 2019). We have extended this to provided a reproducible framework based only on drone remotely‐sensed information; integrating RGB, multispectral and TIR imagery to evaluate how terrain attributes drive biocrust distribution. In this study, the fine spatial resolution of UAV‐based multispectral imagery allowed us to produce very accurate maps of dryland biocrusts, with a higher degree of detail compared to the ones produced in previous studies using airborne and satellite imagery (e.g., Panigada et al., 2019; Rodríguez‐Caballero et al., 2014; Weber et al., 2008). The high accuracy reached in discriminating between typical semi‐arid environmental components (i.e., vascular vegetation, biocrust with different composition and soils) reinforces the ability of the SVM algorithm to distinguish between similar spectral classes (Plaza et al., 2009) and of the CR algorithm's ability to improve identification of biocrusts (e.g., Blanco‐Sacristán et al., 2019; Panigada et al., 2019; Román et al., 2019). Although differences in the absorption feature caused by chlorophyll at ~660 nm between biocrusts and bare soils are subtle (Weber & Hill, 2016), we showed that they can be detected with multispectral data. CR_red_ allowed us to improve the classification results by exploiting the differences between the biocrust classes.

It should be noted that UAV data collection took place during the wet season, when the water content in biocrusts is high and pigments are metabolically active (Weber & Hill, 2016). This enhances the differences in the spectral properties of biocrusts and improves their identification (Blanco‐Sacristán et al., 2019). While the classifications from the multispectral images allowed mapping of the different environmental components of the study area, the values of ATI derived from TIR imagery correlated very highly with the soil moisture measured in the field. We demonstrated that the correlation between ATI and soil moisture is also maintained when biocrust cover is present. To our knowledge, these are the first maps of ATI derived from UAV in drylands with presence of biocrust cover. Previous studies have used hyperspectral thermal sensors to discriminate different types of biocrusts from bare sand based on emissivity values and to characterize biocrusts' maturity (Rozenstein & Karnieli, 2015). In this study the ATI maps derived from TIR imagery were used to estimate how the soil moisture affects biocrust type and distribution, but ATI maps could also be used to better understand water distribution in drylands, especially when there is controversy about the final role of biocrust covers on soil water balance due to their influence on several hydrological processes and soil properties with antagonist effects (Chamizo et al., 2016). We found that topographic characteristics can partially explain the soil moisture gradient estimated by ATI (ATI and PSIR are inversely correlated with *r* ^2^ = 0.46). This moisture gradient promotes a diversification of ecological niches that also explains the distribution pattern of biocrust communities. However, long‐term studies are still necessary to better understand the dynamics of soil moisture and the feedback effects on biocrust cover, and to try to decouple the biocrust effect on water from the water effect on biocrusts, which are strongly interrelated. ATI could be a useful variable to expand our knowledge in this direction.

The UAV RGB images were used to produce fine DTMs from which terrain attributes were derived and utilized to evaluate their impact on soil properties. The mean error of these DTMs was lower than 1 cm, which is comparable to previous studies applying SfM techniques for topography reconstruction (Bakker & Lane, 2017; Smith & Vericat, 2015). These results, together with the rough and steep topography of the study areas, suggest that these models were not affected by systematic error. We found that PSIR, ATI and Northernness were the terrain attributes mostly related to soil properties. These attributes explained in similar magnitudes the distribution of SolSal, OrgCarb, P_tot_ and N_tot_. Increased shadows and higher soil moisture, indicated by low PSIR and high ATI, are related to a higher content of soil nutrients, as shown in plots located in Zones 2 and 4, predominantly east‐ and north‐facing plots, respectively. Increased shadows and soil moisture control the survival and activity of microorganisms (Borken & Matzner, 2009; Drenovsky et al., 2004) and lead to better nutrient cycling and higher activity of microbial communities (e.g., Xue et al., 2018). This increased moisture favours not only the preservation of OrgCarb and its association with other mineral components (Plaza et al., 2013), but also the presence of vegetation and moss‐dominated biocrusts on the most humid slopes. Conversely, plots from Zones 1 and 3 presented the highest PSIR, which determines the lower content of nutrients (lower microbial activity‐lower nutrients) and increased content of SolSal, which is observed in other areas with high evapotranspiration rates (Rodríguez‐Caballero et al., 2019).

The variance in soil properties not explained by the terrain attributes is probably explained by the presence of vegetation and biocrust cover. Vegetation patches enhance the accumulation of water and nutrient capture, which increase biological activity under and close to their cover (e.g., Okin et al., 2015). In addition, vegetation increases shadows which promotes microbial activity (e.g., Huang et al., 2015; Xue et al., 2018) and thus soil nutrients content. *Macrochloa tenacissima* in particular, the dominant species in the study area, modifies stocks of soil carbon and organic matter (e.g. Gauquelin et al., 1996; Kaouthar & Chaieb, 2009; Maestre et al., 2001). While its effect on nirogen (N) content is less clear, some studies have found negative effects on this parameter (Armas & Pugnaire, 2011) while others have described positive effects (Castillo‐Monroy et al., 2010) when compared to non‐vegetated surfaces.

Together with the effect of vegetation on soil properties, biocrusts have been found to take up significant amounts of atmospheric C and N by photosynthesis and N fixation (Elbert et al., 2012). They are therefore an important pool and source of organic inputs into the soil in drylands (Castillo‐Monroy et al., 2011; Chamizo et al., 2012b; Concostrina‐Zubiri et al., 2013). Furthermore, biocrusts strengthen soil structure by interacting with mineral particles and forming aggregates (Belnap & Lange, 2013; Eldridge & Leys, 2003), thus protecting soil from C loss. Biocrusts promote microbial community growth where they appear and the increased shadows created by vegetation increase this growth indirectly (Huang et al., 2015). However, the role of biocrusts on soil nutrients content depends on biocrust composition and patch‐size distribution (e.g.,Bowker et al., 2013; Delgado‐Baquerizo et al., 2015; Sedia & Ehrenfeld, 2006). For example, mosses have greater photosynthetic capacity compared to other types of biocrust (Weber & Hill, 2016), and thus can incorporate higher levels of C. In addition, positive relationships between N and the presence of mosses have already been observed (Delgado‐Baquerizo et al., 2016). This might partially explain the increased soil nutrients content in the north‐ and east‐facing plots in the study area, where mosses appear to dominate plant interspaces.

Variations in biocrust distribution and composition in dryland biomes are primarily controlled by climatic differences (Bowker et al., 2016). However, vegetation and terrain properties generate variations from these climatic controls and modify the local distribution of nutrients in dryland landscapes (e.g., Manzoni et al., 2006; Puigdefábregas, 2005; Puigdefábregas & Sánchez, 1996). Thus, the different sensitivity of biocrusts to the distribution of resources conditions the composition of these ecosystems (Bowker et al., 2016; Durham et al., 2018; Rodríguez‐Caballero et al., 2019). The RDA conducted using data from 1147 points showed that *Fulgensia spp*. presented a strong positive relationship with unstable zones (high values of elevation and LSF). *Fulgensia* spp. has already been observed in unstable terrains several times, highlighting the pioneering behaviour of this genus (Cantón et al., 2020; Miralles et al., 2020; Rodríguez‐Caballero et al., 2013). Mosses, typically found in shaded areas (i.e. *Pleurochaete squarrosa* and *Syntrichia ruralis*), dominated areas with low PSIR, high ATI, and a high presence of vascular vegetation, where soils were richer in organic carbon and nutrients. A positive plant‐biocrust relationship is common for bryophytes (Zhou et al., 2020) and close correspondence with *Macrochloa tenacissima* presence has already been observed (Martínez‐Sánchez et al., 1994).

The RDA showed a lower explanatory power of the terrain attributes regarding *Tortula revolves*, a moss that develops in arid environments, and BLM class dominated by the association of *Diploschistes diacapsis* and *Squamarina lentigera* (BLM). They appeared in more stable zones (i.e., low values of elevation and LSF), as already observed in geographic areas with similar characteristics (e.g., Ladrón de Guevara et al., 2018) that have high solar radiation and soils rich in soluble salts. In particular, *Squamarina lentigera* can be physiologically adapted to light‐exposed environments, and requires high temperatures for optimal photosynthesis while being well hydrated (Lange et al., 1997). Nevertheless, *Diploschistes diacapsis,* the dominant species of BL, was also found in areas with high LSF. This species is very versatile and can adapt its physiology depending on where it grows (Pintado et al., 2005). We observed that *Diploschistes diacapsis* can develop directly on outcrop rocks with very few millimetres of soil beneath them in areas where gypsum substrates with fine soil texture favour stability, but where water availability is scarse. In these areas with outcrop rock, high concentrations of gypsum, soluble salts and higher solar radiation (i.e., high values of PSIR), *Diploschistes diacapsis* is more adapted to establish than other lichens, representing here the early stage of biocrust development.

We found instead mixed patches of lichens and moss (BLM) (thought to be of the last stages of development) in the left axis of the RDA plot, where water availability is still scarces but the terrain presentes lower elevation and LSF and less concentrations of gypsum. Moss‐dominated biocrusts become dominant in areas with higher soil moisture and north‐facing areas, where soil and micro‐environmental conditions are less selective. In the Tabernas desert (southern Spain, Almería; Rodríguez‐Caballero et al., 2019), a nearby site with 220 mm of mean annual rainfall, similar lichen‐dominated biocrusts appear in north‐faced slopes (low evapotranspiration, low PSIR), while in our study area they appear in south‐facing slopes (high evapotranspiration, high PSIR). Biocrust distribution in drylands might be more constrained by the effect of terrain attributes on local hydric availability, rather than rainfall water inputs. For this reason, it is difficult to make generalizations in the development of biocrusts' communities and developing monitoring methodologies that allow up‐scaling local relationships of biocrusts with the terrain is key for dryland ecology.

## 5 CONCLUSIONS

The synergistic information obtained from RGB, multispectral and thermal UAV imagery has allowed us to better understand the complex factors influencing the distribution of dryland ecosystem components. We produced accurate, high‐resolution maps that can help to up‐scale the local effect of biocrust components on ecosystem functioning by improving the accuracy of erosion and infiltration modelling. These aspects of dryland ecology are key to understanding the distinctive role that biocrust‐dominated surfaces have on water runoff and erosion depending on the predominant type of biocrust (e.g., Rodríguez‐Caballero et al., 2015; Wang et al., 2017). Producing high‐resolution maps will help to monitor these communities in space and time, a key task to understand the compositional changes they are already experiencing due to climate change (Escolar et al., 2012; Ladrón de Guevara et al., 2018).

Since field‐based data collection has many drawbacks, mainly related to time and costs (Palmer et al., 2002), exploiting UAV‐based methodologies will help to build standardized procedures in dryland monitoring programmes, while providing very detailed information of these environments. These are needed because drylands may be characterized by different structural and functional organization and present a wide spectrum of compositions, depending on local climate and terrain properties. Our results have shown some discrepancies compared to the traditional biocrust development models, in which more advanced stages (i.e., mixed patches of lichens and moss) are assumed to appear only in stable soils. Thus, we suggest integrating more traditional approaches with new methodologies such as the one developed in this study, which can provide the very fine spatial resolution maps of dryland composition and terrain attributes needed for a detailed description and monitoring of these complex and threatened ecosystems.

## CONFLICT OF INTEREST

The authors declare there is no conflict of interest.

## Supporting information


**Figure S1.** Daily precipitation (P; mm) and minimum and maximum air temperatures (T min and T max, respectively; °C) registered in the study area during 2018, the year when the field campaign took place. The red line evidence the day when the drone flights were conducted.
**Table S1.** Mean emissivity value in the thermoMAP range (8.5–13.5 µm) of typical soil and biocrusts of the study area.
**Table S2.** Confusion matrices of the classification of both study areas. Top: confusion matrix of Area A. Bottom: confusion matrix of Area B. BL: bright lichens; BLM: bright lichens and moss; Fulg: *Fulgensia* spp. and moss; GreenVeg: green vegetation; DryVeg: dry vegetation.
**Table S3.** Explained variance of individual factors in the Redundancy Analysis (RDA) of the coverage of biocrust. Df: degrees of freedom. ATI: apparent thermal inertia; TWI: topographic wetness index; LSF: length slope factor; Veg: vegetation; Soil: bare soil; PSIR: potential incoming solar radiation. ***: *p*‐value < 0.001.

## Data Availability

The data that support the findings of this study are available from the corresponding author upon reasonable request.
