# Supplementary material for: UAV RGB, thermal infrared and multispectral imagery used to investigate the control of terrain on the spatial distribution of dryland biocrust
Source: Earth Surf Process Landf. 2021 Aug 10;46(12):2466–84. doi: 10.1002/esp.5189 (PMC8518773; doi:10.1002/esp.5189)
Supplement: Supplementary file 1 — Figure S1. Daily precipitation (P; mm) and minimum and maximum air temperatures (T min and T max, respectively; °C) registered in the study area during 2018, the year when the field campaign took place. The red line evidence the day when the drone flights were conducted. Table S1. Mean emissivity value in the thermoMAP range (8.5–13.5 µm) of typical soil and biocrusts of the study area. Table S2. Confusion matrices of the classification of both study areas. Top: confusion matrix of Area A. Bottom: confusion matrix of Area B. BL: bright lichens; BLM: bright lichens and moss; Fulg: Fulgensia spp. and moss; GreenVeg: green vegetation; DryVeg: dry vegetation. Table S3. Explained variance of individual factors in the Redundancy Analysis (RDA) of the coverage of biocrust. Df: degrees of freedom. ATI: apparent thermal inertia; TWI: topographic wetness index; LSF: length slope factor; Veg: vegetation; Soil: bare soil; PSIR: potential incoming solar radiation. ***: p‐value < 0.001. [file ESP-46-2466-s001.docx]

**UAV-based RGB, thermal infrared and multispectral imagery to investigate the terrain control on the spatial distribution of dryland biocrust**

*Javier Blanco-Sacristán, Cinzia Panigada, Rodolfo Gentili, Giulia Tagliabue, Roberto Garzonio, M. Pilar Martín, Mónica Ladrón de Guevara, Roberto Colombo, Thomas P.F. Dowling, Micol Rossini*

**Supplementary Material**


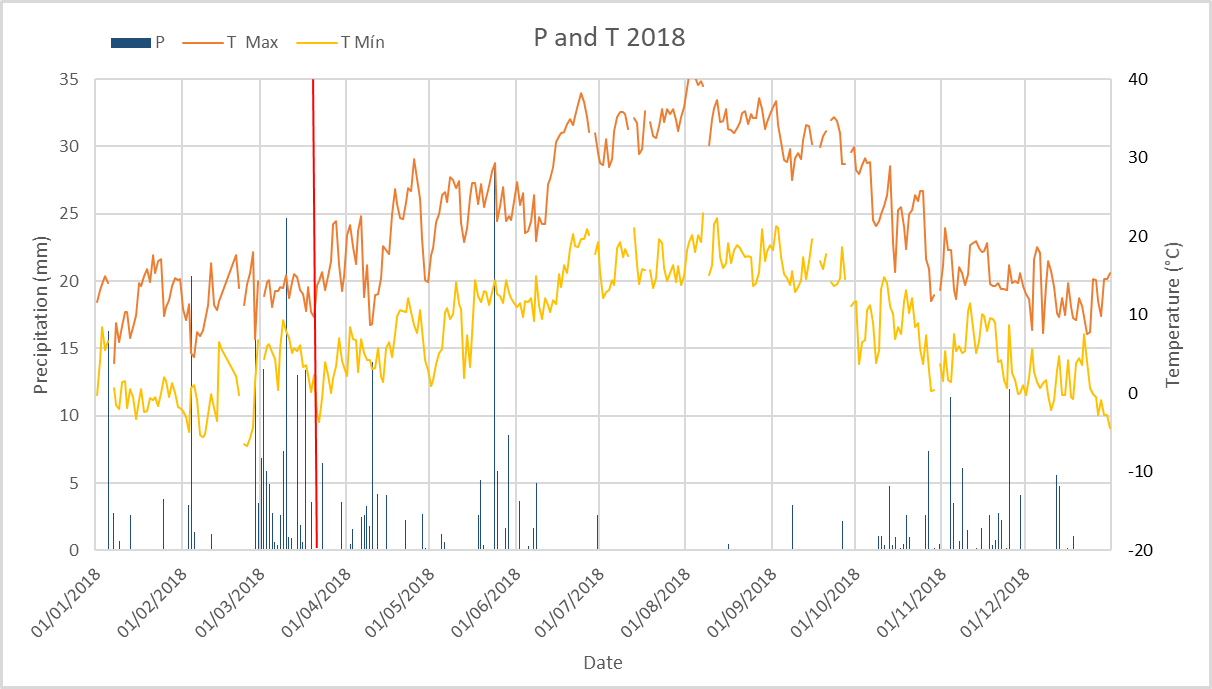


**Figure S1.** Daily precipitation (P; mm) and minimum and maximum air temperatures (T min and T max, respectively; ºC) registered in the study area during 2018, the year when the field campaign took place. The red line evidence the day when the drone flights were conducted.

**Laboratory measurement of thermal emissivity**

Six samples collected from the field were analysed in the King’s College London laboratory using a Bruker Vertex 70 FTIR spectrometer. The spectrometer is modified with an external integrating sphere of diameter 150 mm coated with a diffusely reflecting gold-coating with a 30 mm sampling port to enable directional-hemispherical reflectance measurements of large, inhomogeneous samples. There are two internal sources (MIR and NIR), an external globar water-cooled MIR source and two detectors (MIR and InGaAs respectively) for consideration over 0.7 – 16 μm. The entire system (including the integrating sphere) is continuously purged with H_2_O- and CO_2_ free air at a flow rate of at least 200 L/h to reduce atmospheric features in the spectra and prevent degradation of the KBr beamsplitter (Langsdale et al., 2021).

Each sample was wetted with 100 ml of deionised water and measured immediately. Repeat measurements of each sample were made in the following time-steps after the first; 1 minute, 1 hour, 3 hours, 12 hours, 24 hours and 48 hours. Three measurements of each sample were made at each timestep; (i) centre of petri dish, (ii) stepped 15 mm to the top and left, and (iii) stepped 15 mm to the bottom and right of the first centre measurement. The stepping was done in order to capture the full variability of the sample. The sample was pressed close to the sampling port each time in order that no ambient radiation contaminated the readings.

See Langsdale et al., (2021) for a full description of the emissivity measurement and validation approach employed by spectrometer. We here used the substitution method, therefore each of the three measurements of a sample at a given timestamp required the following measurement sequence; gold reference panel, sample, gold reference panel, open port and then gold reference panel once more.

**Table S1.** Mean emissivity value in the thermoMAP range (8.5-13.5 μm) of typical soil and biocrusts of the study area.

| Sample type | Dry | Wet |
| --- | --- | --- |
| Bare soil | 0.977 ± 0.018 | 0.980 ± 0.011 |
| Pleurochaete squarrosa | 1.000 ± 0.004 | 0.997 ± 0.003 |
| Syntrichia ruralis | 0.996 ± 0.004 | 0.988 ± 0.005 |
| Fulg | 0.985 ± 0.004 | 0.994 ± 0.003 |
| BLM | 0.987 ± 0.005 | 0.993 ± 0.003 |
| BL | 0.986 ± 0.007 | 0.992 ± 0.004 |

**References**

Langsdale, M. F., Wooster, M., Harrison, J. J., Koehl, M., Hecker, C., Hook, S. J., Abbott, E., Johnson, W.R., Maturilli, A., Poutier, L., Lau, I. C., Brucker, F. (2021). Spectral emissivity (SE) measurement uncertainties across 2.5–14 μm derived from a round-robin study made across international laboratories. Remote Sensing, 13(1), 102.

**Table S2.** Confusion matrices of the classification of both study areas. Top: confusion matrix of Area A. Bottom: confusion matrix of Area B. BL: bright lichens; BLM: bright lichens and moss; Fulg: *Fulgensia* spp. and moss; GreenVeg: green vegetation; DryVeg: dry vegetation.

| Study area A |  | Field data (%) | | | | | |
| --- | --- | --- | --- | --- | --- | --- | --- |
|  |  | Soil | DryVeg | GreenVeg | BL | BLM | Moss |
|  | Soil | 82.22 | 0 | 0 | 2.22 | 14.22 | 0 |
|  | DryVeg | 0 | 83.78 | 6.17 | 0 | 0 | 10.35 |
| Classified data (%) | GreenVeg | 0 | 6.76 | 93.83 | 0 | 0 | 0 |
|  | BL | 10 | 0 | 0 | 86 | 0 | 0 |
|  | BLM | 7.78 | 0 | 0 | 11.78 | 74.67 | 8.37 |
|  | Moss | 0 | 9.46 | 0 | 0 | 11.11 | 81.28 |

| Study area B |  | Field data (%) | | | | | |
| --- | --- | --- | --- | --- | --- | --- | --- |
|  |  | Soil | DryVeg | GreenVeg | BL | Fulg | Moss |
|  | Soil | 94.52 | 0 | 0 | 2.22 | 4.44 | 0 |
|  | DryVeg | 0 | 89.41 | 0 | 0 | 0 | 0 |
| Classified data (%) | GreenVeg | 0 | 2.35 | 97.59 | 0 | 0 | 1.18 |
|  | BL | 1.37 | 0 | 0 | 95.56 | 3.33 | 0 |
|  | Fulg | 4.11 | 0 | 0 | 2.22 | 92.22 | 8.24 |
|  | Moss | 0 | 8.24 | 2.41 | 0 | 2 | 90.59 |

**Table S3.** Explained variance of individual factors in the Redundancy Analysis (RDA) of the coverage of biocrust. Df: degrees of freedom. ATI: apparent thermal inertia; TWI: topographic wetness index; LSF: length slope factor; Veg: vegetation; Soil: bare soil; PSIR: potential incoming solar radiation. ***: *p*-value < 0.001.

|  | Df | Variance | F | Pr(>)F |
| --- | --- | --- | --- | --- |
| ATI | 1 | 0.344 | 130.175 | 0.001 *** |
| TWI | 1 | 0.085 | 32.064 | 0.001 *** |
| LSF | 1 | 0.237 | 89.644 | 0.001 *** |
| Veg | 1 | 0.094 | 35.486 | 0.001 *** |
| Soil | 1 | 0.0724 | 27.379 | 0.001 *** |
| PSIR | 1 | 0.529 | 200.070 | 0.001 *** |
| Elevation | 1 | 0.258 | 97.637 | 0.001 *** |
| Residual | 1139 | 3.011 |  |  |
